# Efficient Recovery of Rare Earth Elements and Zinc from Spent Ni–Metal Hydride Batteries: Statistical Studies

**DOI:** 10.3390/nano12132305

**Published:** 2022-07-05

**Authors:** Ahmed R. Weshahy, Ayman A. Gouda, Bahig M. Atia, Ahmed K. Sakr, Jamelah S. Al-Otaibi, Aljawhara Almuqrin, Mohamed Y. Hanfi, M. I. Sayyed, Ragaa El Sheikh, Hend A. Radwan, Fatma S. Hassen, Mohamed A. Gado

**Affiliations:** 1 Department of Chemistry, Faculty of Science, Zagazig University, Zagazig 44519, Egypt; ahmed.r.weshahy@gmail.com (A.R.W.); aymangouda77@gmail.com (A.A.G.); ragaaelsheikh@hotmail.com (R.E.S.); 2 Nuclear Materials Authority, El Maadi, Cairo P.O. Box 530, Egypt; dr_bahig.atia@yahoo.com (B.M.A.); mokhamed.khanfi@urfu.ru (M.Y.H.); noda_gomaa71@yahoo.com (H.A.R.); fatmasalem7788@gmail.com (F.S.H.); 3 Department of Civil and Environmental Engineering, Wayne State University, 5050 Anthony Wayne Drive, Detroit, MI 48202, USA; 4 Department of Chemistry, College of Science, Princess Nourah Bint Abdulrahman University, P.O. Box 84428, Riyadh 11671, Saudi Arabia; jsalotabi@pnu.edu.sa; 5 Department of Physics, College of Science, Princess Nourah Bint Abdulrahman University, P.O. Box 84428, Riyadh 11671, Saudi Arabia; ahalmoqren@pnu.edu.sa; 6 Institute of Physics and Technology, Ural Federal University, St. Mira, 19, 620002 Yekaterinburg, Russia; 7 Department of Physics, Faculty of Science, Isra University, Amman 11622, Jordan; dr.mabualssayed@gmail.com

**Keywords:** rare earth elements (REEs), cerium, zinc, recovery, Ni–metal hydride (Ni–MH) batteries

## Abstract

Considering how important rare earth elements (REEs) are for many different industries, it is important to separate them from other elements. An extractant that binds to REEs inexpensively and selectively even in the presence of interfering ions can be used to develop a useful separation method. This work was designed to recover REEs from spent nickel–metal hydride batteries using ammonium sulfate. The chemical composition of the Ni–MH batteries was examined. The operating leaching conditions of REE extraction from black powder were experimentally optimized. The optimal conditions for the dissolution of approximately 99.98% of REEs and almost all zinc were attained through use of a 300 g/L (NH_4_)_2_SO_4_ concentration after 180 min of leaching time and a 1:3 solid/liquid phase ratio at 120 °C. The kinetic data fit the chemical control model. The separation of total REEs and zinc was conducted under traditional conditions to produce both metal values in marketable forms. The work then shifted to separate cerium as an individual REE through acid baking with HCl, thus leaving pure cerium behind.

## 1. Introduction

Rare earth elements (REEs) refer to the lanthanides (57–71) besides scandium (Sc) and yttrium (Y). REEs are involved in a broad range of products [1,2,3,4]. Electric vehicles and wind energy are becoming more widespread. This means there is a higher demand for REEs. There is a possibility that their supply could be disrupted due to geological paucity, extraction difficulties, and national dependence on their use. Thus, they are considered to be truly essential raw commodities that are riskier to obtain and have a greater economic impact on Europe’s monetary union [5]. With regards to the development of sources beyond primary sources, the use of REEs promotes their long-term sustainability. Spent battery consumption is expected to be the fastest-growing category of WEEE. Rechargeable batteries have a life cycle of about 1000 cycles. Every 3–5 years, the cycle turns into municipal solid waste. Extensive application of lithium-ion batteries (LIBs) results in greater battery usage. As a result, there has been a dramatic decrease in the usage of nickel–metal hydride (Ni–MH) batteries; thus, the production of Ni–MH batteries has decreased. In addition, spent Ni–MH batteries pose an environmental hazard that can be managed through effective management, making the recycling of metals from these devices an important topic of study [6,7]. The Ni–MH battery is not rechargeable for further use; the electrode is made of, e.g., a porous 30.0% rare earth metals alloy such as the La10.5Ce4.3Pr0.5Nd1.4Ni60.0Co12.7Mn5.9Al4.7 alloy (where the numbers represent atomic percentages of elements) [8]. For the metallurgical recovery of REEs from these batteries, the number of metallurgical operations must be considered. Poor energy efficiency and severe environmental pollution are characteristics of spent Ni–MH battery pyro-metallurgical processes. Electric arc furnaces and non-ferrous smelters have low waste concentrations; therefore, REEs are commonly reverted to the slag phase to deal with the wastes there. This can make economical recovery more difficult. Decreased energy consumption and low investment costs are among the key advantages of hydrometallurgical processing, in which several mineral acids are used to leach Ni–MH scrap [9,10,11,12,13,14].

Large concentrations of REEs and nickel in its metallic formula mean that, with organic acids, the REEs and nickel almost completely dissipate. Because organic acids are less effective than inorganic acids at leaching Ni and Co, the low efficiency of metal recovery as a whole is apparent. Sulfuric acid is more commonly found in household and commercial applications. It is an alternative treatment to hydrochloric acid for Ni–MH power batteries because it is inexpensive and good for the environment. Since it is resistant to oxidation and corrosion and has a boiling point of 78 °C, it is not a good choice for a leaching agent, for example. Ni becomes oxidized when exposed to sulfuric acid. At the same temperature, rare earth elements and cobalt dissolve in water [15,16,17]. Due to the low amount of energy needed and minimal greenhouse gas emissions, hydrometallurgical processing for metal recovery is advantageous. Leaching with HCl [12] and H_2_SO_4_ [18] followed by a mixture of separation procedures is used to treat spent Ni–MH batteries [19]. Other metal ions were separated by solvent extraction in the majority of prior studies after H_2_SO_4_ leaching, REE precipitation, and solvent extraction. Under the aforementioned circumstances, neodymium (Nd) was recovered in high concentrations at 99.1%, while Sm was recovered at 98.4%, Pr was recovered in high concentrations at 95.54%, and Ce was recovered in high concentrations at 89.0% [20]. Separate research into selective metal extraction was undertaken primarily to reduce acid usage compared to conventional leaching methods such as direct sulfuric acid leaching. According to Meshram et al., the acid baking process changes the metal’s reactivity and generates sulfates, breaching the impenetrable barrier [21].

Several studies have focused on selectively recovering REEs. Expended nickel–metal hydride battery acid was leached in two stages, followed by acidic pH precipitation. In the first stage of traditional leaching, 2.5 M of H_3_PO_4_ for 60 min at 25 °C was employed to eliminate large amounts of heavy metals such as Ni, Co, Cd, and Zn, and more than 90.0% of the rare earth metals were transformed into insoluble phosphate precipitates (PO_4_). The second-stage process included subcritical water extraction and a low H_2_SO_4_ concentration was utilized to extract all of the rare earth elements. As the concentration of leachant acid increased, the leaching efficiency of REEs increased, and 100% of the REEs, Co, Ni, and Mn were leached using 1.0 M of H_2_SO_4_ (20 g/L) for 30 min at 125 °C. At low pH (0.5–2.0), REEs precipitate as NaREE(SO_4_).2H_2_O and separate from other metals. Acidification at pH 1.0 resulted in the formation of hexagonal rod crystals composed of 82.59% La, 85.77% Nd, and 90.0% Ce [22]. Diphenyl phosphate (DBP) has a high precipitation efficacy for RE^3+^ ions; it precipitates La^3+^, Nd^3+^, Pr^3+^, and Ce^3+^, which can attain 97.84, 99.70, 100, and 100%, respectively. Nonetheless, less than 1.75% of Co^2+^, Ni^2+^, and Mn^2+^ ions were precipitated. DBP can also be recycled easily and reused. After five cycles, the precipitation efficiency of DBP did not change [23].

On the other hand, ammonium sulfate plays a foremost role in the selective extraction of REEs and Zn from various media. Ammonium compounds have become one of the most popular leachants for extracting rare earth elements in recent years. Moldoveanu and Papangelakis used (NH_4_)_2_SO_4_ to leach and recover REE ions [24,25]. Kim et al. also used a 1.0% concentration of (NH_4_)_2_SO_4_ to leach REEs from Vietnamese ore [26]. The use of ammonium sulfate has the advantages of reduced reagent use and increased final product purity [27].

In this work, suggested procedures for the efficient recovery of REEs from spent nickel–metal hydride batteries were applied to leach all REEs and almost all zinc using ammonium sulfate. The work then shifted to individually separating cerium from the REE cake using HCl. Moreover, statistical studies related to the leaching process were conducted.

## 2. Materials and Methods

### 2.1. Preparation of Spent Nickel–Metal Hydride (Ni–MH) Batteries

**Figure 1:**
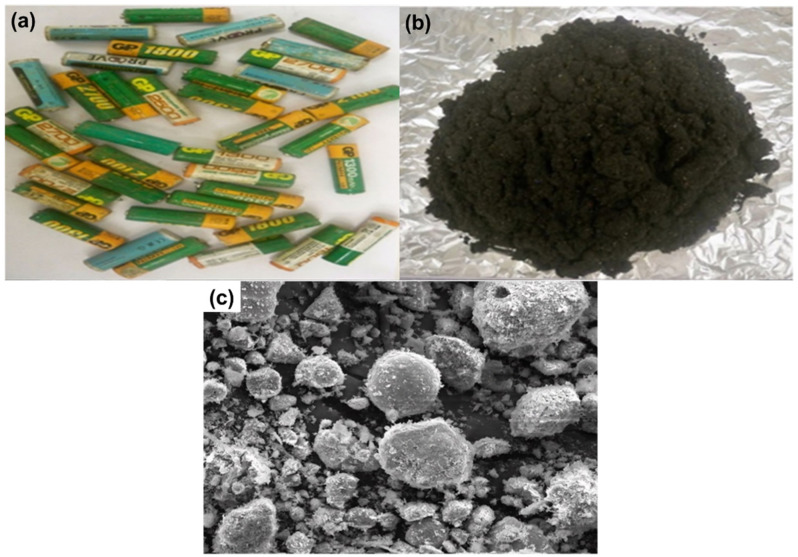
(**a**) Spent nickel–metal hydride (Ni–MH) batteries; (**b**) Black powder of Ni–MH batteries; (**c**) SEM of the obtained black powder of Ni–MH batteries.

The samples recycled in this work were AA- and AAA-type cylindrical spent (Ni–MH) batteries produced in Japan (Figure 1a). These batteries were collected from local scraps. To remove the steel case with its associated battery electrode and the nylon separator from the expended batteries, they were ground and crushed in a scutter cutter using deionized water as the medium. Table 1 shows the battery components of the batteries, their weight, and their proportion. The electrode material spread in the deionized water as the heavier particles, primarily the steel casing, dropped to the bottom. The lighter nylon separator then floated away. After the removal of the float fraction (nylon) from the solution, the suspended fraction (black powder) was wet screened. The electrode powder (approximately 73.17 g) was accumulated and washed repeatedly with water in order to eliminate any possible metals (e.g., Na and K) that could behave as an activator for double sulfate precipitation, which causes REE damage throughout the acid dissolution process, from the working electrolyte [28]. After that, the battery powder was dried at 80 °C for 24 h. The obtained black powder was subjected to calcination at 550 °C to remove organic matter [29]. After milling and calcining, the weight of the battery black powder was detected. The powder was analyzed for its chemical composition, particle size, and morphological characteristics.

### 2.2. Apparatus and Chemicals

Many different analytical techniques were used to characterize the raw or fresh original materials, the leaching residues, and the resulting products. ICP–OES (Ultima Expert, Horiba Scientific, Kyoto, Japan) was used to measure the concentration of REEs and heavy metals in the solution. The morphologies of the black powder and the final products (REEs as double sulfates) were identified using a scanning electron microscope (SEM–EDX) (LEO 1450VP, Carl Zeiss Microscopy GmbH, Jena, Germany). X-ray diffraction (XRD) (D8 Discover Family, Bruker, Billerica, MA, USA) was utilized to investigate the structure of the crystalline materials. Furthermore, the chemicals employed in the leaching and precipitation of the products were analytical-grade compounds. NaOH, (NH_4_)_2_SO_4_, H_2_SO_4_, HCl, HNO_3_, and H_2_O_2_ were supplied by Sigma-Aldrich, St. Louis, MO, USA.

## 3. Results and Discussion

### 3.1. Pre-Treatment and Characterization of the Battery Powder

In the beginning, the black powder of the Ni–MH battery was treated with H_2_O_2_ to prevent iron leaching during the leaching process (Figure 1b); H_2_O_2_ acts as an oxidizing agent for the oxidation of Fe(II) to Fe(III) ions. This was performed by treating the battery powder with 30.0% H_2_O_2_ using a 4:1 S/L ratio at 100 °C for 2 h. The obtained data showed that no ferrous iron was present in the sample and all iron content was ferric. This may have been due to the fact that ferrous iron reacts easier with ammonium sulfate to form Mohr’s salt [30].

REEs, including lanthanum (La), cerium (Ce), neodymium (Nd), and praseodymium (Pr), are essential to the nickel–metal hydride (Ni–MH) battery supply chain since they are found in high concentrations in Ni–MH batteries. REEs may have a secondary resource in the form of recovered metals from work on spent Ni–MH batteries. The REEs were collected from discarded Ni–MH batteries via selective alkaline leaching with ammonium sulfate solution. Almost all the REEs were leached from the electrode active material of the discarded Ni–MH batteries. The chemical composition of the Ni–MH batteries is tabulated in Table 2. The SEM of the obtained black powder is shown in Figure 1c. The latter shows the morphology of the black powder, which clearly demonstrates scattered metal aggregates with a high degree of nickel-based phase intercalation.

### 3.2. Leaching of REEs from Spent Ni–MH Batteries

#### 3.2.1. Influence of (NH_4_)_2_SO_4_ Concentration

**Figure 2:**
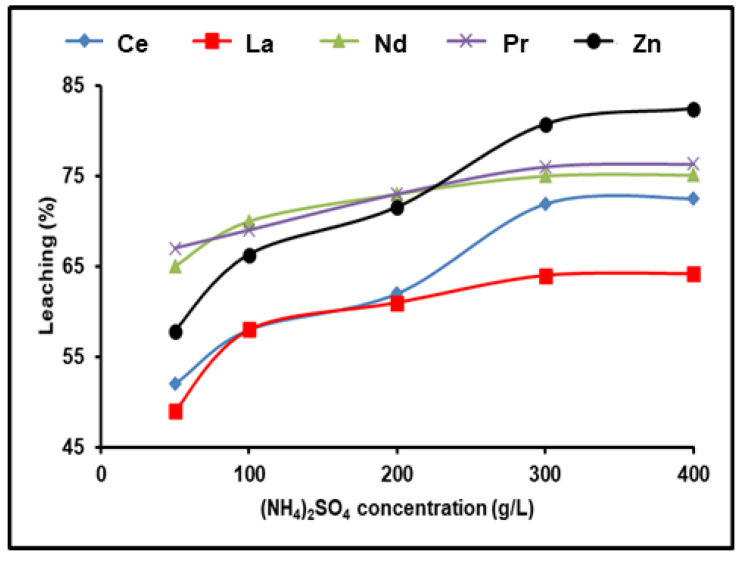
Influence of ammonium sulfate concentration on the dissolution of REEs and Zn from spent Ni–MH battery powder (T: 90 °C, t: 120 min, 1:2 (S/L) ratio).

The leaching procedure was conducted using 1.0 g of Ni–MH black powder, 120 min, and 500 rpm at 90 °C to investigate how much of the spent Ni–MH powder leached REEs at various concentrations of (NH_4_)_2_SO_4_ (50–400 g/L). Using an agitator set to 500 rpm kept the particles in suspension and reduced the lixiviant diffusion layer that formed around them. We found that the dissolution of rare earth ions increased progressively as the concentration of (NH_4_)_2_SO_4_ was raised, up to 300 g/L. The efficiency dissolution process of all REEs remained practically constant even after increasing the concentration of (NH_4_)_2_SO_4_. As a result, 300 g/L of (NH_4_)_2_SO_4_ was the optimum concentration for leaching in this experiment. With 300 g/L (NH_4_)_2_SO_4_, 98% Nd, 76.3% Pr, 71.9% Ce, 64.2% La, and 80.7% Zn were leached out in 120 min (Figure 2).

#### 3.2.2. Influence of Time

**Figure 3:**
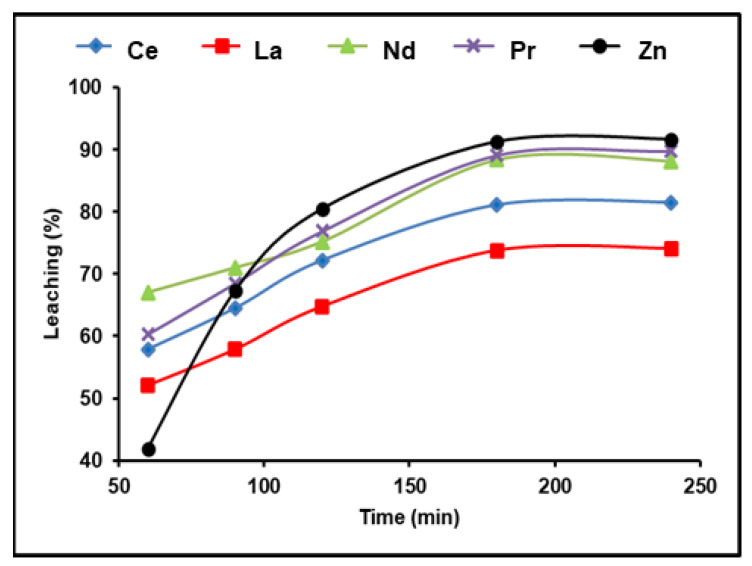
Influence of time on the dissolution of REEs and Zn from spent Ni–MH battery powder (300 g/L (NH_4_)_2_SO_4_, T: 90 °C, 1:2 (S/L) ratio).

A 300 g/L (NH_4_)_2_SO_4_ and 1:2 (S/L) ratio solution was used to test the time effect on rare earth metal leaching at 90 °C and a pulp density of 100 g/L. The obtained results in Figure 3 demonstrate that the amount of REEs recovered improved as the amount of leaching time increased. A La leaching efficiency of 74.1%was achieved after 180 min; further leaching efficiencies were found as 88.4% for Nd, 89.1% for Pr, and 81.5% for Ce. Any additional increment in the leaching time after 180 min had no influence on the leaching efficiency of the five elements. We found that 180 min of leaching yielded the greatest amount of dissolution of the REEs and also attained about 92% leaching efficiency for zinc.

#### 3.2.3. Influence of The Solid/Liquid (S/L) Ratio

**Figure 4:**
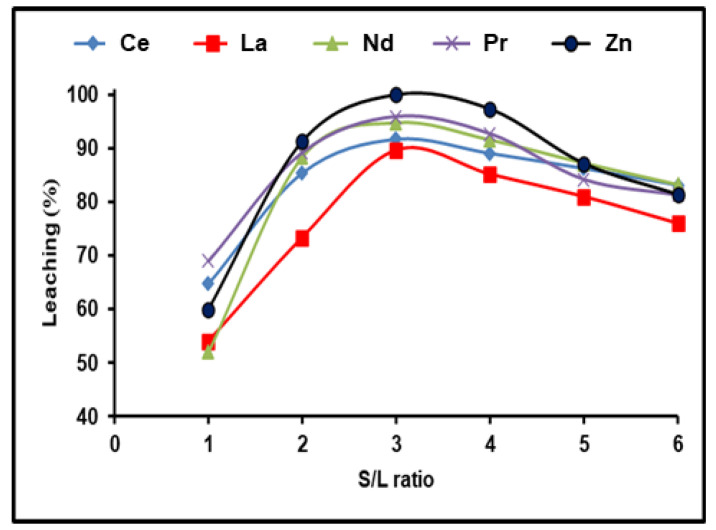
Influence of S/L ratio on the dissolution of REEs and Zn from spent Ni–MH battery powder (300 g/L (NH_4_)_2_SO_4_, T: 90 °C, t: 180 min).

REE dissolving efficiencies were studied in the range between 1:1 and 1:6 depending on the solid/liquid ratio. For these trials, the stable dissolution conditions included 300 g/L (NH_4_)_2_SO_4_ and an incubation time of 180 min with a dissolving temperature of 90 °C. REE leaching efficiency was found to increase with increasing the S/L phase ratio to 1:3, followed by a reduction as the S/L ratio increased from 1:4 to 1:6 (Figure 4). This may be because REEs precipitate as mono-sulfates. It is clear that all the available REEs reacted with ammonium sulfate in the solution until achieving equilibrium, after which any excess of (NH_4_)_2_SO_4_ had no effect on the REE leaching efficiency. As a result, a S/L phase ratio of 1:3 yielded the highest possible dissolution efficiencies of 91.7, 89.4, 94.7, and 95.9% for Ce, La, Nd, and Pr, respectively, and almost 100% for Zn.

#### 3.2.4. Influence of Temperature

**Figure 5:**
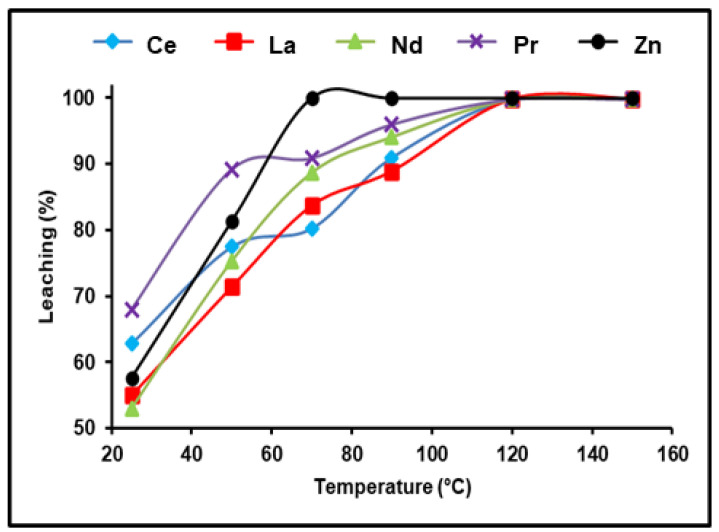
Influence of temperature on the dissolution of REEs and Zn from spent Ni–MH battery powder (300 g/L (NH_4_)_2_SO_4_, t: 180 min, 1:3 (S/L) ratio).

The effect of temperature on the solubility of various REEs extant in spent Ni–MH powder was investigated throughout a temperature ranging between 25 and 150 °C. The obtained data in Figure 5 show temperature’s substantial influence on obtaining the highest REE leaching efficiency. At ambient temperature and experimental conditions of 300 g/L of (NH_4_)_2_SO_4_, 180 min, and a 1:3 S/L phase ratio, 62.81, 55.3, 53.4, and 68.7% of Ce, La, Nd, and Pr were leached, respectively. The dissolution efficiency reached its maximum when raising the leaching temperature to 120 °C and any further raise in temperature did not demonstrate any significant improvement in the leaching of the REEs. Consequently, we found that the satisfactory dissolution temperature of Ce, La, Nd, and Pr was 120 °C under the previously mentioned conditions.

According to a prior examination of the effectiveness of leaching REEs from spent NiMH battery powder using ammonium sulfate, the optimal dissolution conditions for dissolving approximately 99.98% of REEs and almost all zinc are as follows: 300 g/L of (NH_4_)_2_SO_4_, 180 min, 1/3 for the S:L phase ratio, and 120 °C.

### 3.3. Dissolution Kinetic Analysis of REEs

Three methods can be used to identify the rate of reaction: chemical reaction at the core surface of the particle, diffusion through the solid product layer, or diffusion via the fluid. The rate of the procedure is dictated by the progress of the slowest phase in succession [31]. In the shrinking core model (SCM), the reactions occur on the solid’s outer layer and decline towards the center. Meanwhile, the fluid–solid reaction models are considered the most influential models that have been applied [32]. In the combination model, the reaction occurs on the outer surface of the solid and declines towards the center, when a solid particle (M) is submerged in a fluid (N) and reacts with the fluid based on the following equation:

N(fluid)+bM(solid)→products

The required time (*t*) for solid reaction can be determined using the next equation if the reaction rate is controlled by fluid N diffusion through the ash layer:

$$1-3{\left(1-x\right)}^{\frac{2}{3}}+2\left(1-x\right)=\frac{6bDC_{o}t}{C_{B}r_{o}^{2}}=K_{1}t$$

where *x* is defined as the fraction of dissolved REEs, *D* (m^2^/s) represents the diffusivity of the REEs from the ash layer, *C_o_* and *C_B_* (mol/L) correspond to fluid concentration outside the particle and the concentration of solid reactant, respectively, *r_o_* (m) is the initial outer radius of the particle, *K*_1_ as well as *K*_2_ both stand for the rate constant, and *t* (min) denotes the solubilization time. If the chemical reaction controls the reaction rate, the equation of the integrated rate is calculated as follows:

$$1-{\left(1-x\right)}^{\frac{1}{3}}+2\left(1-x\right)=\frac{bk_{d}C_{o}t}{C_{B}r_{o}}=K_{2}t$$

**Figure 6:**
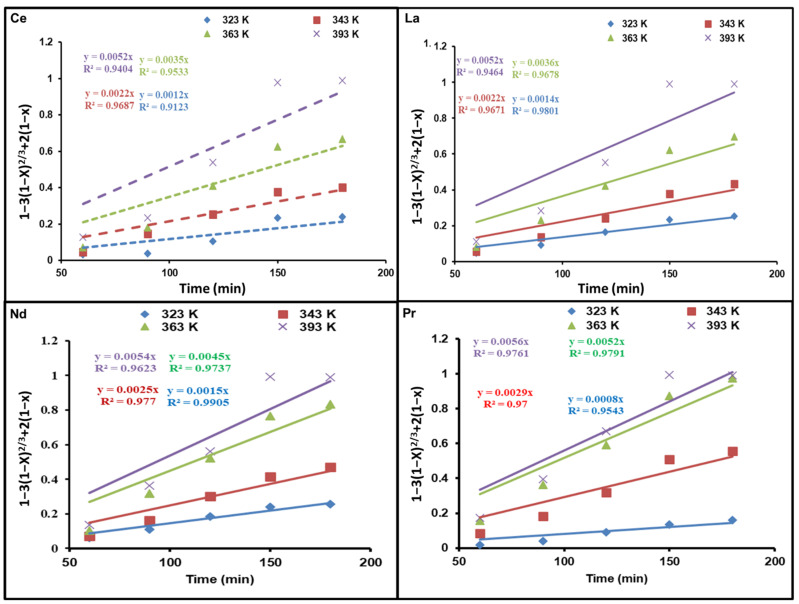
Influence of dissolution time on fraction of REEs dissolved, 1 − 3(1 − *x*)^2/3^ + 2(1 − *x*), at different temperatures.

**Figure 7:**
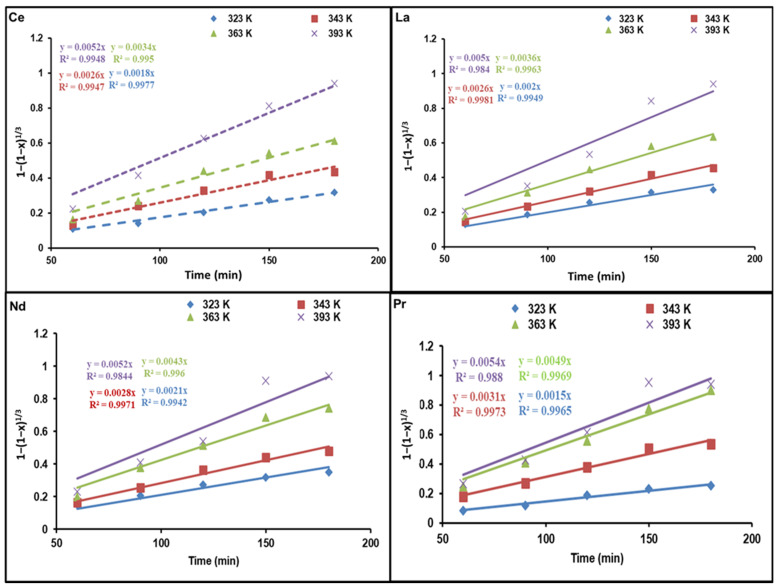
Influence of dissolution time on fraction of REEs dissolved, 1 − (1 − *x*)^1/3^, at different temperatures.

Figure 6 and Figure 7 demonstrate diagrams of 1 − 3(1 − *x*)^2/3^ + 2(1 − *x*) as well as 1 − (1 − *x*)^1/3^ against dissolution time; they indicate how temperature affects REE reaction kinetics. The plots show straight lines for Ce, La, Nd, and Pr at various temperatures. As presented in Figure 6, the kinetic data are not a good fit for the diffusion control model. Nevertheless, the calculated R^2^ values in Figure 7 are above those of the diffusion control model, indicating that the kinetic results match well with the chemical control model.

**Figure 8:**
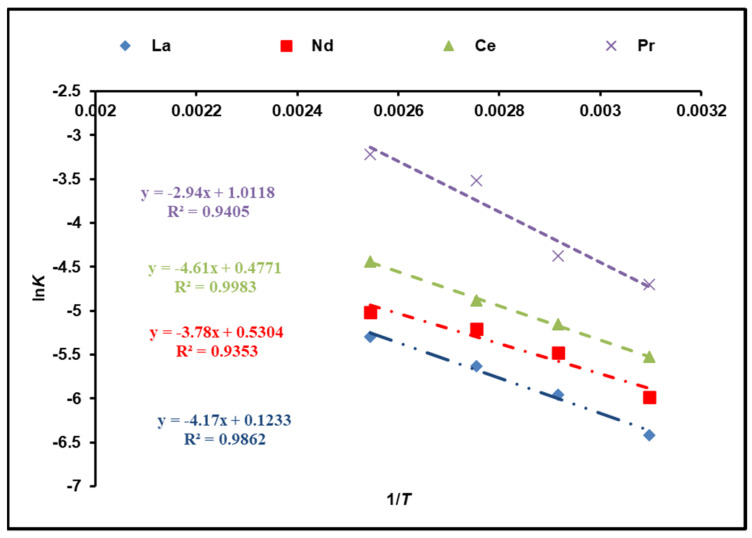
Activation energy calculations of REEs from Arrhenius plot.

The activation energy of the dissolution reaction (*E_a_*) is calculated using the Arrhenius equation and the rate constants of *K_chem_*:

$$\ln K=\frac{-E_{a}}{RT}+\ln A$$

where *E_a_* (kJ/mol) is attributed to the activation energy, *A* corresponds to the Arrhenius constant, and *R* is 8.314 J/mol K. Figure 8 illustrates a plot of ln*K* against 1/*T*. According to the calculations, the activation energies of cerium, lanthanum, neodymium, and praseodymium in the temperature range of 323–393 K are 38.33, 34.67, 31.43, and 24.44 kJ/mol, respectively, which was determined from the slope. As a result, the obtained results are rather suitable for the model of a shrinking core (SCM) with a chemical reaction as a rate-determining step [33,34].

### 3.4. Regression and Correlation Results between Leaching Factors and REEs

The Pearson correlation is a multiple linear regression model that measures the strength of the linear relationship between two variables. It has a value between −1 to 1, with a value of −1 meaning a total negative linear correlation between leaching factors and elements. In this study, it was applied in order to investigate the relationship between different leaching factors ((NH_4_)_2_SO_4_ concentration, time, S/L ratio, and temperature) of REEs (Ce, La, Nd, and Pr) and Zn. All factors were considered as explanatory variables along with the leaching efficiencies (%) of REEs as dependent variables. The results of the regression model demonstrate that there was a positive relationship with significant significance between the (NH_4_)_2_SO_4_ conc and all of the explanatory variables (REE leaching efficiency, %). This was inferred from the R^2^ value, which indicates the magnitude of the relationship between the set of predictors in the regression and the outcome variable; and the beta coefficient, which represents the degree of variation in the outcome variable for every 1 unit of change in the predictor variable, with its associated *p*-value and Pearson correlation; alongside covariance, which indicates the value of the reverse relationship. The explanatory variables explain more than 75.0% of the variations in leaching efficiency, signifying that there was a very strong relationship between all REE leaching efficiency variations and the explanatory variables ((NH_4_)_2_SO_4_ concentration, time, and temperature). This was with the exception of Ce and temperature, which had a moderate positive relationship with no significant relationship.

The results also show a strong negative relationship with significant significance between the S/L ratio and La with Nd, but the other REEs had a moderate negative relationship with no statistical significance with the S/L ratio.

Variance inflation factors (VIFs) are used to measure how much the variance of the estimated regression coefficients is inflated as compared to when the predictor variables are not linearly related. VIFs are used to verify how much amount of multicollinearity (correlation between predictors) exists in a regression analysis. The value of the VIF of the model was 1, indicating the non-existence of multicollinearity problems (Table 3 and Table 4). Thus, the results indicate the following regression model equations.

**Figure 9:**
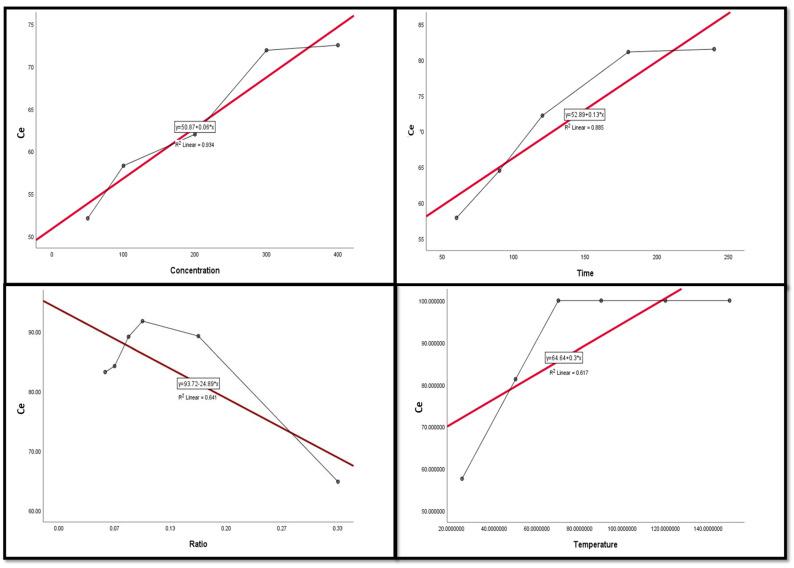
The scatter plot with linear regression fit for Ce leaching efficiency, %, and factors.

**Figure 10:**
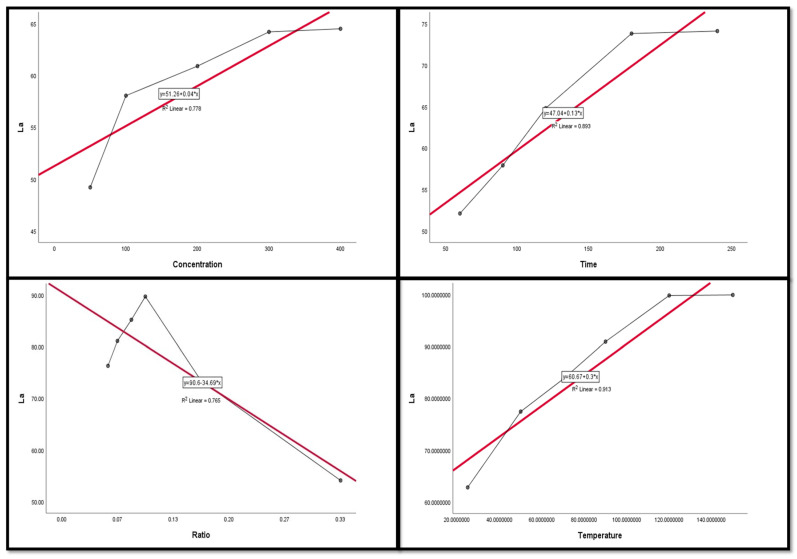
The scatter plot with linear regression fit for La leaching efficiency, %, and factors.

**Figure 11:**
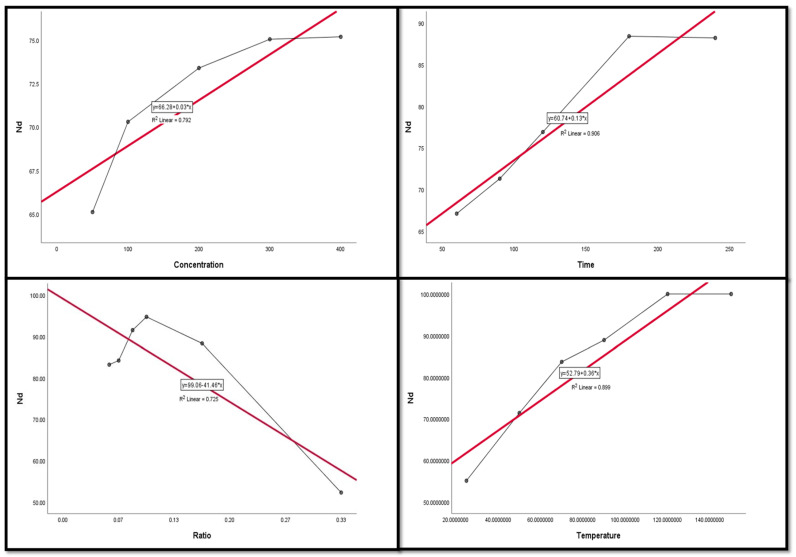
The scatter plot with linear regression fit for Nd leaching efficiency, %, and factors.

**Figure 12:**
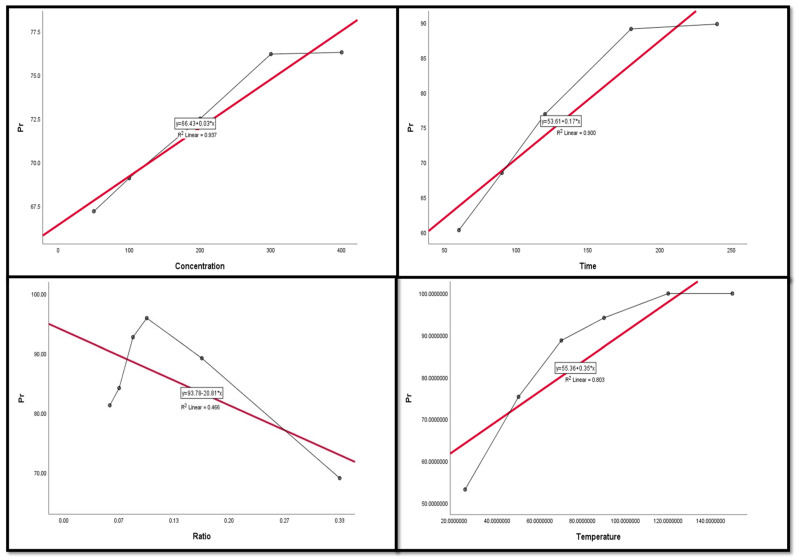
The scatter plot with linear regression fit for Pr leaching efficiency, %, and factors.

**Figure 13:**
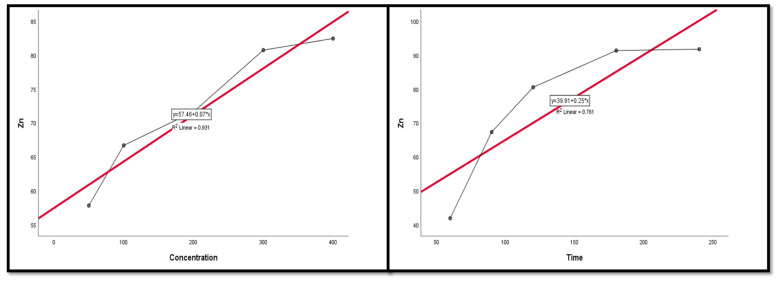
The scatter plot with linear regression fit for Zn leaching efficiency, %, and factors.

Figure 9, Figure 10, Figure 11, Figure 12 and Figure 13 display the scatter plot of the linear regression fit for the leaching efficiency of the five elements against different factors, which confirmed the same results with regards to where the relationship was positive and strong with the (NH_4_)_2_SO_4_ concentration, leaching time, S/L phase ratio, and temperature. It is noteworthy to mention that at the start point there was no leaching and that if any variable was set to equal 0, there was no possible leaching. Additionally, it is of note that the (NH_4_)_2_SO_4_ concentration and time of Ce, Pr, and Zn leaching had a stronger positive effect than temperature, while the impacts of temperature and time on Nd and La were stronger than that of (NH_4_)_2_SO_4_ concentration. On the other hand, it is clear that there was a strong negative relationship between the solid/liquid phase ratio and the leaching efficiency of the REE metals.

### 3.5. Separation of Zinc and REEs Products

**Figure 14:**
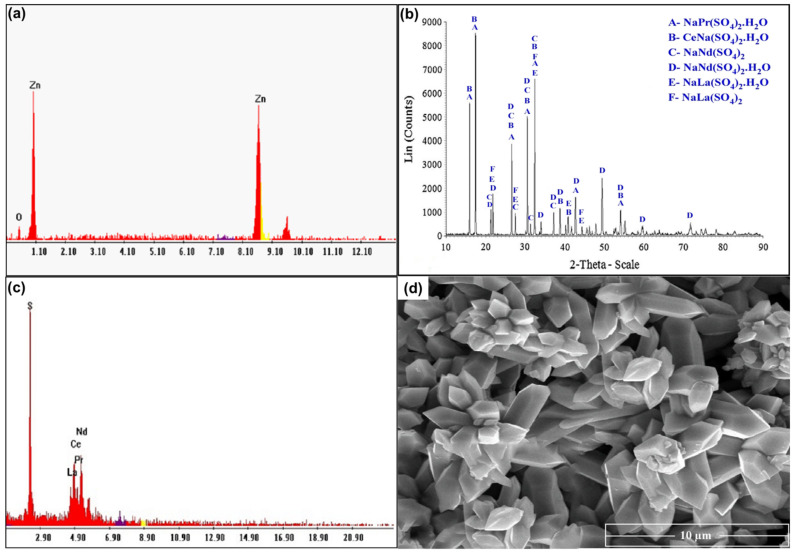
(**a**) EDX spectrum of ZnO product; (**b**) XRD spectrum of RE sulfate salts; (**c**) EDX spectrum of REE product; (**d**) SEM image of REE product.

For studying the recovery procedures for REEs from the spent Ni–MH battery powder, proper ammonium sulfate leach liquor was prepared using the previously studied optimum leaching conditions. Leached REEs can be recovered through precipitation as their hydroxides or else via selective REE precipitation as sulfates or oxalates. Leached Zn and REEs can be recovered via co-precipitation as their hydroxides at a pH of 8.0. Zn(OH)_2_ can be precipitated from its salt solution by adding a suitable base. An excess of alkali base quickly dissolves Zn(OH)_2_ as sodium zincates such as NaZn(OH)_3_ and Na_2_Zn(OH)_4_ [35]. The Zn was removed from the solution using CO_2_ gas to precipitate it as ZnO, as seen in Figure 14a. After removing ZnO, the rare earth elements were precipitated as double insoluble sulfate salts using sulfuric acid, which was validated by XRD, as presented in Figure 14b. There were several phases detected in the solution such as the following salts: NaCe(SO_4_)_2_.H_2_O, NaPr(SO_4_)_2_.H_2_O, NaNd(SO_4_)_2_.H_2_O, NaNd(SO_4_)_2_, NaLa(SO_4_)_2_H_2_O, and NaLa(SO_4_)_2_. Additionally, the EDX spectrum confirmed the structure of REEs, as given in Figure 14c. The concentration of elements in the product was determined to be 45.7% S, 17.5% La, 14.83% Ce, 11.17% Nd, and 10.8% Pr. The SEM image in Figure 14d shows a hexagonal shape of RE sulfate salts, which are fully developed. It shows well-structured and tiny particles of 5–6 µm in length and 1–2 µm in width.

### 3.6. Separation of Ce(IV) Individually

The dried REE powder was subjected to another leaching process in order to separate cerium individually from the REE mixture (Ce, Ln, Nd, and Pr) using hydrochloric acid [36,37]. For the chemical separation of Ce(IV) from the REEs in the generated mixture (Ce, Ln, Nd, and Pr), the trivalent lanthanides were dissolved in a 10.0% HCl solution, whilst the insoluble Ce(IV) was left behind. The efficiency of Ce(IV) separation was primarily influenced by the pH of the chloride leach liquor and the time required for the REEs combination to dissolve.

#### 3.6.1. Influence of pH

**Figure 15:**
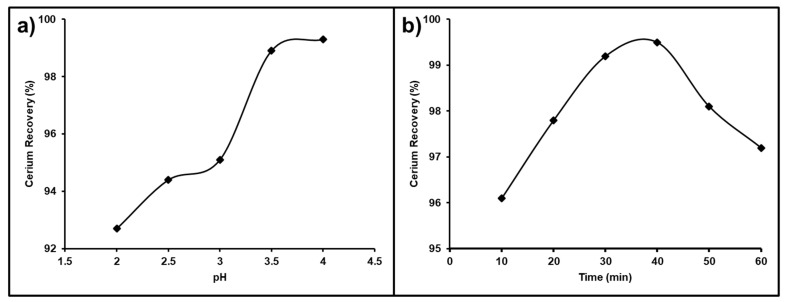
(**a**) Influence of pH; (**b**) influence of time on Ce separation from the REEs.

The solution pH is the most important and effective parameter affecting cerium purity and recovery from a REE mixture. The pH value of chloride solutions was studied in the range from 2.0 to 4.0 that was used for the leaching process with stirring at ambient temperature. The obtained results presented in Figure 15a show that the recovery of Ce(IV) improved gradually as the pH increased from 92.7 to 98.1%; as a result, a chloride leach liquor pH of 3.5 was selected as the sufficient pH value for the recovery of cerium, which was not leached with other associated REEs.

#### 3.6.2. Influence of Time

The impact of the stirring time on the cerium separation efficiency from the REE mixture was studied between 10–60 min (Figure 15b). It was observed that by raising the leaching time from 10 to 40 min, the leaching of the other associated trivalent REEs in the chloride solution at a pH value of 3.5 was increased so that the best dissolution time for cerium recovery from the REEs mixture was 40 min.

#### 3.6.3. Cerium Oxide Separation

**Figure 16:**
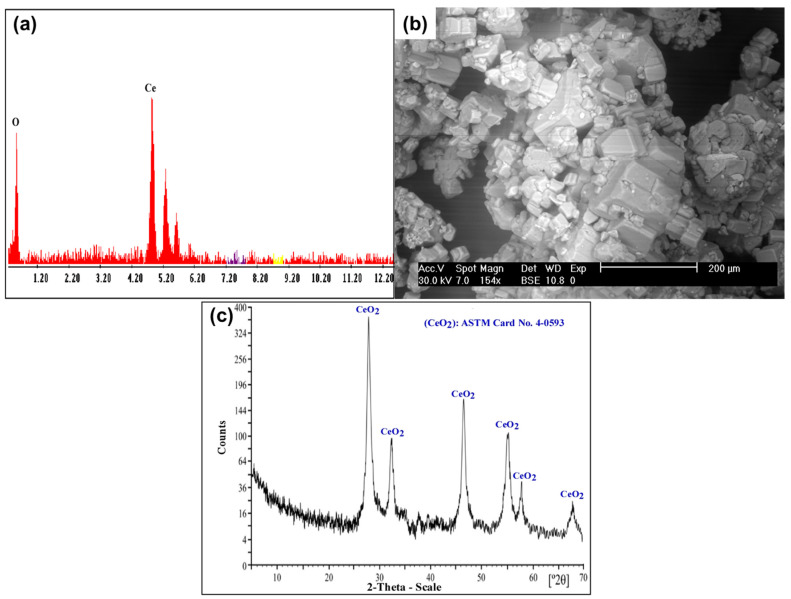
(**a**) EDX spectrum of CeO_2_ product; (**b**) SEM image of CeO_2_ product; (**c**) XRD spectrum of CeO_2_ product.

The yellowish insoluble Ce(IV) was separated from the chloride leach liquor. Subsequently, several washes with distilled H_2_O were performed to eliminate the excess of Cl^−^ ions from the yellowish Ce(IV) precipitate. The obtained product was confirmed using SEM–EDX and XRD analysis, as shown in Figure 16.

### 3.7. Flowsheet

**Figure 17:**
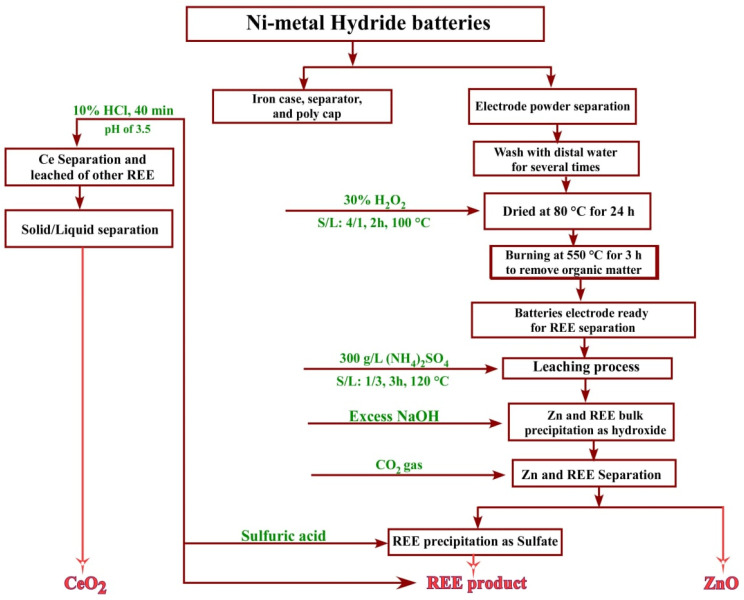
Flowsheet for the recovery of REEs from Ni–metal hydride batteries.

Figure 17 displays the flowsheet of the recovery process of rare earth elements, zinc, and cerium(IV) from spent Ni–metal hydride batteries.

## 4. Conclusions

The recovery of REEs and zinc from spent nickel hydride batteries was performed using ammonium sulfate as a leachant; the optimal conditions of alkali baking were attained using a 300 g/L (NH4)_2_SO_4_ concentration, after 180 min of leaching time and a 1:3 solid/liquid phase ratio at 120 °C. The dissolution percentage was 99.98% in both the REEs and zinc; we found the kinetic data to be in good agreement with the chemical control model. After a nearly complete leaching of both the zinc and REEs, the separation between them through conventional techniques was nearly completed. Subsequently, Ce ions were individually separated from the total REE cake using 10.0% of HCl at pH 3.5 for 40 min at ambient temperature. Several washes with distilled H_2_O were performed to remove the excess of Cl^−^ ions from the yellowish Ce(IV) precipitate. Both XRD and SEM–EDX techniques were applied to detect the pure cerium(IV) product.

## Figures and Tables

**Table 1:** Weight and proportions of the spent nickel–metal hydride battery parts after physical separation.

Components	Weight (g)	Proportion (%)
Iron case	23.68	22.77
Separator	6.88	6.62
Poly cap	0.3	0.29
Electrode powder	73.14	70.32

**Table 2:** Chemical characterization of spent Ni–MH battery powder.

Element	Ni	Co	Zn	Mn	Fe	Ce	La	Pr	Nd	Others
Wt. (%)	45.813	4.225	1.56	2.875	6.3	6.225	5.502	1.9199	2.438	7.282

**Table 3:** Regression model equations.

(NH_4_)_2_SO_4_ Concentration and S/L Ratio	Time and Temperature
Ce leaching efficiency, % = 50.87 + 0.06 × (NH_4_)_2_SO_4_ conc.	Ce leaching efficiency, % = 52.89 + 0.13 × Time
Ce leaching efficiency, % = 93.77−24.66 × S/L ratio	Ce leaching efficiency, % = 64.64 + 0.3 × Temp.
La leaching efficiency, % = 51.26 + 0.778 × (NH_4_)_2_SO_4_ conc.	La leaching efficiency, % = 47.04 + 0.13 × Time
La leaching efficiency, % = 90.62−34.25 × S/L ratio	La leaching efficiency, % = 60.67 + 0.3 × Temp.
Nd leaching efficiency, % = 66.28 + 0.03 × (NH_4_)_2_SO_4_ conc.	Nd leaching efficiency, % = 60.74 + 0.13 × Time
Nd leaching efficiency, % = 99.17 − 41.14 × S/L ratio	Nd leaching efficiency, % = 52.79 + 0.3 × Temp.
Pr leaching efficiency, % = 66.43 + 0.03 × (NH_4_)_2_SO_4_ conc.	Pr leaching efficiency, % = 53.61 + 0.17 × Time
Pr leaching efficiency, % = 93.72 − 20.36 × S/L ratio	Pr leaching efficiency, % = 55.36 + 0.35 × Temp.
Zn leaching efficiency, % = 57.46 + 0.07 × (NH_4_)_2_SO_4_ conc.	Zn leaching efficiency, % = 39.91 + 0.25 × Time
Zn leaching efficiency, % = 122.72 − 34.24 × S/L ratio	Zn leaching efficiency, % = 71.88 + 0.22 × Temp.

**Table 4:** Regression and correlation results between leaching factors and REEs.

Regression and Correlation Results		Ce	La	Nd	Pr	Zn
(NH_4_)_2_SO_4_ concentration (g/L)	Pearson correlation	0.966 **	0.882 *	0.890 *	0.968 **	0.965 **
	Sig. (*p*-value)	0.007	0.048	0.043	0.007	0.008
	Covariance	1219.250	791.625	540.100	569.250	1405.750
	R^2^	0.934	0.778	0.792	0.937	0.931
	Beta (unstandardized)	0.06	0.04	0.03	0.03	0.07
	N	5	5	5	5	5
Time (min)	Pearson correlation	0.941 *	0.945 *	0.952 *	0.949 *	0.872
	Sig. (*p*-value)	0.017	0.015	0.013	0.014	0.054
	Covariance	701.850	662.100	667.200	881.550	1310.100
	R^2^	0.885	0.893	0.906	0.900	0.761
	Beta (unstandardized)	0.13	0.13	0.13	0.17	0.25
	N	5	5	5	5	5
S/L ratio	Pearson correlation	−0.788	−0.858 *	−0.840 *	−0.664	−0.742
	Sig. (*p*-value)	0.063	0.029	0.037	0.150	0.091
	Covariance	−2.422	−3.363	−4.040	−2.000	−3.362
	R^2^	0.621	0.736	0.705	0.441	0.551
	Beta (unstandardized)	−24.66	−34.25	−41.14	−20.36	−34.24
	N	6	6	6	6	6
Temperature (°C)	Pearson correlation	0.786	0.955 **	0.948 **	0.896 *	0.859 *
	Sig. (*p*-value)	0.064	0.003	0.004	0.016	0.028
	Covariance	629.143	627.127	759.577	745.683	469.635
	R^2^	0.617	0.913	0.899	0.803	0.739
	Beta (unstandardized)	0.300	0.3	0.36	0.35	0.22
	N	6	6	6	6	6

*: correlation is significant at the 0.05 level; **: correlation is significant at the 0.01 level.

## Data Availability

Not applicable.

## References

[1] Allam E.M., Lashen T.A., Abou El-Enein S.A., Hassanin M.A., Sakr A.K., Hanfi M.Y., Sayyed M.I., Al-Otaibi J.S., Cheira M.F. (2022). Cetylpyridinium Bromide/Polyvinyl Chloride for Substantially Efficient Capture of Rare Earth Elements from Chloride Solution. Polymers.

[2] Sakr A.K., Mohamed S.A., Mira H.I., Cheira M.F. (2018). Successive leaching of uranium and rare earth elements from El Sela mineralization. J. Sci. Eng. Res..

[3] Allam E.M., Lashen T.A., Abou El-Enein S.A., Hassanin M.A., Sakr A.K., Cheira M.F., Almuqrin A., Hanfi M.Y., Sayyed M.I. (2022). Rare Earth Group Separation after Extraction Using Sodium Diethyldithiocarbamate/Polyvinyl Chloride from Lamprophyre Dykes Leachate. Materials.

[4] Sakr A.K., Cheira M.F., Hassanin M.A., Mira H.I., Mohamed S.A., Khandaker M.U., Osman H., Eed E.M., Sayyed M.I., Hanfi M.Y. (2021). Adsorption of Yttrium Ions on 3-Amino-5-Hydroxypyrazole Impregnated Bleaching Clay, a Novel Sorbent Material. Appl. Sci..

[5] Sethurajan M., van Hullebusch E.D., Fontana D., Akcil A., Deveci H., Batinic B., Leal J.P., Gasche T.A., Ali Kucuker M., Kuchta K. (2019). Recent advances on hydrometallurgical recovery of critical and precious elements from end of life electronic wastes—A review. Crit. Rev. Environ. Sci. Technol..

[6] Marra A., Cesaro A., Belgiorno V. (2019). Recovery opportunities of valuable and critical elements from WEEE treatment residues by hydrometallurgical processes. Environ. Sci. Pollut. Res. Int..

[7] Mahmoud N.S., Atwa S.T., Sakr A.K., Abdel Geleel M. (2012). Kinetic and thermodynamic study of the adsorption of Ni (II) using Spent Activated clay Mineral. N. Y. Sci. J..

[8] Meng T., Young K.-h., Koch J., Ouchi T., Yasuoka S. (2016). Failure Mechanisms of Nickel/Metal Hydride Batteries with Cobalt-Substituted Superlattice Hydrogen-Absorbing Alloy Anodes at 50 °C. Batteries.

[9] Pietrelli L., Bellomo B., Fontana D., Montereali M.R. (2002). Rare earths recovery from NiMH spent batteries. Hydrometallurgy.

[10] Larsson K., Ekberg C., Ødegaard-Jensen A. (2013). Dissolution and characterization of HEV NiMH batteries. Waste Manag..

[11] Binnemans K., Jones P.T., Blanpain B., Van Gerven T., Yang Y., Walton A., Buchert M. (2013). Recycling of rare earths: A critical review. J. Clean. Prod..

[12] Tzanetakis N., Scott K. (2004). Recycling of nickel–metal hydride batteries. I: Dissolution and solvent extraction of metals. J. Chem. Technol. Biotechnol..

[13] Rodrigues L.E.O.C., Mansur M.B. (2010). Hydrometallurgical separation of rare earth elements, cobalt and nickel from spent nickel–metal–hydride batteries. J. Power Sources.

[14] Resende L.V., Morais C.A. (2010). Study of the recovery of rare earth elements from computer monitor scraps—Leaching experiments. Miner. Eng..

[15] Gasser M.S., Aly M.I. (2013). Separation and recovery of rare earth elements from spent nickel–metal-hydride batteries using synthetic adsorbent. Int. J. Miner. Process..

[16] Zhao Z., Qiu Z., Yang J., Lu S., Cao L., Zhang W., Xu Y. (2017). Recovery of rare earth elements from spent fluid catalytic cracking catalysts using leaching and solvent extraction techniques. Hydrometallurgy.

[17] Meshram P., Somani H., Pandey B.D., Mankhand T.R., Deveci H., Abhilash (2017). Two stage leaching process for selective metal extraction from spent nickel metal hydride batteries. J. Clean. Prod..

[18] Innocenzi V., Vegliò F. (2012). Recovery of rare earths and base metals from spent nickel-metal hydride batteries by sequential sulphuric acid leaching and selective precipitations. J. Power Sources.

[19] Tanong K., Tran L.-H., Mercier G., Blais J.-F. (2017). Recovery of Zn (II), Mn (II), Cd (II) and Ni (II) from the unsorted spent batteries using solvent extraction, electrodeposition and precipitation methods. J. Clean. Prod..

[20] Meshram P., Pandey B.D., Mankhand T.R. (2016). Process optimization and kinetics for leaching of rare earth metals from the spent Ni–metal hydride batteries. Waste Manag..

[21] Meshram P., Abhilash, Pandey B.D., Mankhand T.R., Deveci H. (2016). Acid baking of spent lithium ion batteries for selective recovery of major metals: A two-step process. J. Ind. Eng. Chem..

[22] Lie J., Liu J.-C. (2021). Selective recovery of rare earth elements (REEs) from spent NiMH batteries by two-stage acid leaching. J. Environ. Chem. Eng..

[23] Zhi H., Ni S., Su X., Xie W., Zhang H., Sun X. (2022). Separation and recovery of rare earth from waste nickel-metal hydride batteries by phosphate based extraction-precipitation. J. Rare Earths.

[24] Moldoveanu G.A., Papangelakis V.G. (2012). Recovery of rare earth elements adsorbed on clay minerals: I. Desorption mechanism. Hydrometallurgy.

[25] Moldoveanu G.A., Papangelakis V.G. (2013). Recovery of rare earth elements adsorbed on clay minerals: II. Leaching with ammonium sulfate. Hydrometallurgy.

[26] Kim J.-A., Dodbiba G., Tanimura Y., Mitsuhashi K., Fukuda N., Okaya K., Matsuo S., Fujita T. (2011). Leaching of Rare-Earth Elements and Their Adsorption by Using Blue-Green Algae. Mater. Trans..

[27] Chi R., Xu Z., Yu J., He Z. (2013). Rare Earth Elements 2013.

[28] Porvali A., Wilson B.P., Lundström M. (2018). Lanthanide-alkali double sulfate precipitation from strong sulfuric acid NiMH battery waste leachate. Waste Manag..

[29] Meshram P., Pandey B.D., Mankhand T.R. (2015). Leaching of base metals from spent Ni–metal hydride batteries with emphasis on kinetics and characterization. Hydrometallurgy.

[30] Gibbs M.M. (1979). A simple method for the rapid determination of iron in natural waters. Water Res..

[31] Karatepe Ö., Yıldız Y., Pamuk H., Eris S., Dasdelen Z., Sen F. (2016). Enhanced electrocatalytic activity and durability of highly monodisperse Pt@PPy–PANI nanocomposites as a novel catalyst for the electro-oxidation of methanol. RSC Adv..

[32] Gado M.A., Atia B.M., Fathy W.M. (2020). Selective recovery of the main metal values from Rosetta monazite mineral concentrate. Int. J. Environ. Anal. Chem..

[33] Levenspiel O. (1972). Chemical Reaction Engineering.

[34] Olanipekun E. (1999). A kinetic study of the leaching of a Nigerian ilmenite ore by hydrochloric acid. Hydrometallurgy.

[35] Cotton F.A., Wilkinson G. (1976). Basic Inorganic Chemistry.

[36] Kedari C.S., Pandit S.S., Ramanujam A. (1999). Studies on the In Situ Electrooxidation and Selective Permeation of Cerium(IV) across a Bulk Liquid Membrane Containing Tributyl Phosphate as the Ion Transporter. Sep. Sci. Technol..

[37] Amer T.E., Abdellah W.M., Abdel Wahab G.M., El-Shahat M.F. (2016). Selective Separation of Yttrium and Cerium(IV) from the Prepared Abu Hamata Lanthanides Cake. Chem. Biomol. Eng..

